# Flexible Biocomposites with Enhanced Interfacial Compatibility Based on Keratin Fibers and Sulfur-Containing Poly(urea-urethane)s

**DOI:** 10.3390/polym10101056

**Published:** 2018-09-21

**Authors:** Ibon Aranberri, Sarah Montes, Itxaso Azcune, Alaitz Rekondo, Hans-Jürgen Grande

**Affiliations:** CIDETEC Research Centre, Paseo de Miramón, 196, 20014 Donostia-San Sebastián, Gipuzkoa, Spain; smontes@cidetec.es (S.M.); iazcune@cidetec.es (I.A.); arekondo@cidetec.es (A.R.); hgrande@cidetec.es (H.-J.G.)

**Keywords:** biocomposites, thermoplastic poly(urea-urethane)s, disulfide bond, chicken feathers, fibres

## Abstract

Feathers are made of keratin, a fibrous protein with high content of disulfide-crosslinks and hydrogen-bonds. Feathers have been mainly used as reinforcing fiber in the preparation of biocomposites with a wide variety of polymers, also poly(urea-urethane)s. Surface compatibility between the keratin fiber and the matrix is crucial for having homogenous, high quality composites with superior mechanical properties. Poly(urea-urethane) type polymers are convenient for this purpose due to the presence of polar functionalities capable of forming hydrogen-bonds with keratin. Here, we demonstrate that the interfacial compatibility can be further enhanced by incorporating sulfur moieties in the polymer backbone that lead to new fiber-matrix interactions. We comparatively studied two analogous thermoplastic poly(urea-urethane) elastomers prepared starting from the same isocyanate-functionalized polyurethane prepolymer and two aromatic diamine chain extenders, bis(4-aminophenyl) disulfide (TPUU-SS) and the sulfur-free counterpart bis(4-aminophenyl) methane (TPUU). Then, biocomposites with high feather loadings (40, 50, 60 and 75 wt %) were prepared in a torque rheometer and hot-compressed into flexible sheets. Mechanical characterization showed that TPUU-SS based materials underwent higher improvement in mechanical properties than biocomposites made of the reference TPUU (up to 7.5-fold higher tensile strength compared to neat polymer versus 2.3-fold). Field Emission Scanning Electron Microscope (FESEM) images also provided evidence that fibers were completely embedded in the TPUU-SS matrix. Additionally, density, thermal stability, and water absorption of the biocomposites were thoroughly characterized.

## 1. Introduction

Exploitation of keratin which is a fibrous structural protein making up feathers, wool [1] or horns [2], is gaining attention in the quest for sustainability and waste reduction. Considering only the enormous amount of feathers produced by the poultry industry worldwide [3], any application that could make the best possible use of chicken feathers (CFs) would support the cause. Total or partial replacement of petroleum derived plastics and composites, e.g., using keratin as reinforcing fiber [4,5] or polymer matrix component [6,7,8], is a technical target with promising environmental benefits. Other compiling technical applications are oil absorbents [9], fertilizers [10] and sizing agent for textile yarns [11].

Cysteine (7.8 mol %), glycine (13.7 mol %), proline (9.8 mol %) and serine (14.1 mol %) are the most repeated amino acids in the keratin structure [12,13]. The cross-linking by disulfide and hydrogen bonds results in fibers that are strong, lightweight and with good thermal and insulating properties [14]. Using CFs as fiber reinforcement has led to the development of lightweight biocomposites with insulating properties [4]. This is a straightforward, relatively cheap and interesting application since the inner hollow structure of the feathers is maintained and exploited with few processing steps.

Within biocomposite materials, poly(urea-urethane) elastomers have attracted less interest than biodegradable thermoplastic materials used as matrices, e.g., polylactic acid (PLA) and polybutylene succinate (PBS). Only few references are found in the literature dealing with the combinations with CFs. A. L. Martinez-Hernández developed porous membranes for diverse applications [15] and G. Ozkoc [16] demonstrated that keratin fibers/poly(urea-urethane) composites could be used as a potential biomaterial for tissue engineering. Additionally, the combination of CFs in elastic foams was motivated by the hypothesis of enhancing the fire-resistance of poly(urea-urethane) as shown by Wrzéniewska-Tosik [17].

**Figure 1:**
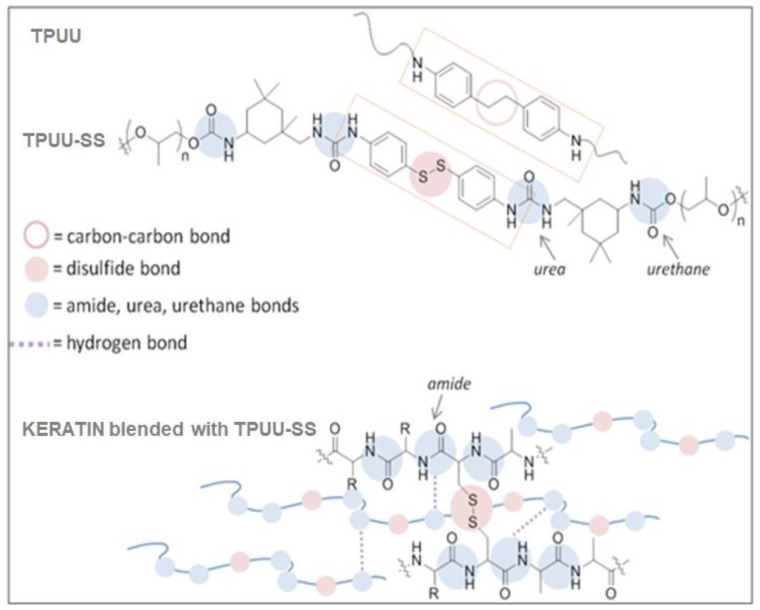
Chemical structure of TPUU, TPUU-SS and keratin.

Thermoplastic poly(urea-urethane) elastomers are versatile block copolymers comprised of alternating soft and hard segments corresponding to polyol and urea and urethane components respectively. Keratin and these elastomers share common structural features that lead to potential chain interactions and surface compatibility (Figure 1). Keratin has amide bonds, whereas poly(urea-urethane) has urea and urethane bonds. These chemical moieties provide amino and carbonyl groups which act as proton-donors and -acceptors, along with other active functional groups of the polymer for establishing hydrogen bonds. It is assumed that this contributes the compatibilization between both surfaces.

Additionally keratin has disulfide bridges between the cysteine amino acids. We hypothesized that elastomers with disulfides on their structure might show even higher compatibility towards this biopolymer since new types of interactions might occur. Sulfur-based chemistry has been extensively used in the field of dynamic polymers [18,19]. In particular, aromatic disulfides undergo chemical exchange at room temperature and lead to the development of poly(urea-urethane)s networks with unprecedented properties such as self-healing and reprocessability [20]. These polymers have to be considered as “living polymers” due to the non-stop reshuffling of the covalent disulfide bonds leading to a constant rearrangement of the polymer segments. Therefore, we expect that this phenomenon might contribute to a better impregnation and mixing of the keratin fibers during the compounding and processing of the biocomposites compared to an analogous sulfur-free elastomer.

Aiming at proving this hypothesis, we have selected two thermoplastic poly(urea-urethane)s with disulfide bonds (TPUU-SS) and without disulfide linkages (TPUU) for the preparation of biocomposites with different weight percentage of CFs. Both thermoplastic materials were made from the same isocyanate-functionalized polyurethane prepolymer and using two different aromatic diamines as chain extenders. Bis(4-aminophenyl) disulfide was used in one case and 4,4′-ethylenedianiline was chosen as the counterpart. The single difference between the two final elastomers is the presence of a reversible aromatic disulfide (–S–S–) or an ethylene (–CH_2_–CH_2_–) repeating unit in the polymer chain. Biocomposites with a range of CF loadings (40, 50, 60 and 75 wt %) were prepared in a torque rheometer and they were further processed as flexible sheets by hot compression. A systematic thermal, mechanical and morphological characterization was carried out to elucidate the main differences between the various formulations.

## 2. Materials and Methods

### 2.1. Materials

**Figure 2:**
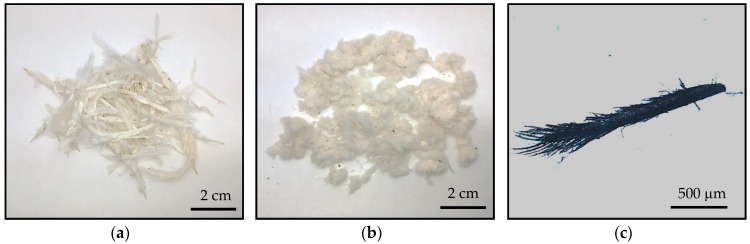
(**a**) Sanitized chicken feathers (CFs), (**b**) ground sanitized CFs and (**c**) 50× magnification of (**b**).

Sanitized chicken feathers were supplied by Grupo SADA (Madrid, Spain). The feathers were washed with industrial alkali detergent and then dried at 60 °C for 24 h. Then, chicken feathers were sterilized in an autoclave with pressurized steam treatment at 126 °C for 30 min, followed by 30 min of drying. Microbiological tests confirmed the absence of pathogens. Finally, sanitized feathers were ground in a universal cutting mill Fritsch Pulverisette 19 (Idar-Oberstein, Germany) at a rotor rotational speed of 2800 rpm and using a sieve insert size of 1 mm. Ground feathers showed a wide size distribution ranging from 100 μm to a few mm (see Figure 2).

Poly(propylene glycol) (PPG) of different molecular weight (*M*_n_ 1000 and *M*_n_ 2000) were purchased from Covestro (Leverkusen, Germany). Isophorone diisocyanate (IPDI), dibutyltin dilaurate (DBTDL), bis(4-aminophenyl) disulfide (4-AFD), tetrahydrofurane (THF) and 4,4′-ethylenedianiline (EDA) were purchased from Sigma-Aldrich (St. Louis, MO, USA).

### 2.2. Synthesis of TPUUs

The route for the synthesis of the TPUUs is described elsewhere [20]. Briefly, bis-isocyanate terminated prepolymers were synthesized from IPDI and PPG (*M*_n_ 1000) and PPG (*M*_n_ 2000) respectively. The bis-isocyanate terminated prepolymers were mixed in a weight ratio of 25/75, *M*_n_ 1000/2000. Then, a concentrated solution of 4-AFD (TPUU-SS) or EDA (TPUU) in tetrahydrofuran was added. The curing was allowed to proceed for 16 h at 60 °C and was monitored by FT-IR spectroscopy. The resulting TPUU was obtained as a transparent elastomeric material.

### 2.3. Biocomposite Preparation

Prior to the blending process of the CFs with the TPUU and TPUU-SS, ground CFs were dried at 80 °C for 6 h. Biocomposites containing 40 (60/40), 50 (50/50), 60 (40/60) and 75 (25/75) wt % of CFs were mixed with TPUU and TPUU-SS (polymer/CF, 100/0) in the torque rheometer HAAKE PolyLab QC (Karlsruhe, Germany) at 150 °C during 10 min. Neat TPUU, neat TPUU-SS, TPUU/CF and TPUU-SS/CF blends were compression-molded in a Vogt 600T laboratory hot press machine (Maschinen + Technik Vogt GmbH, Möhnesee, Germany) into sheets of 90 × 90 × 2.1 mm^3^. Consolidation was carried out at 150 °C for 5 min. Thermogravimetric analysis (TGA) analyses of the blends were performed previously in order to determine thermal stability and set the consolidation conditions (see Section 3.2).

### 2.4. Characterization of the Biocomposites

#### 2.4.1. Density of the Biocomposites

The density of the biocomposites was determined experimentally according to the ISO 9427 [21]. Three rectangular samples of each composite with a known volume were weighed and the density was determined as the ratio of the mass to volume. The average and standard deviation were reported.

#### 2.4.2. Thermogravimetric Analysis (TGA)

The thermal stability was measured by thermogravimetric analysis using a TGA Q500 (TA Instruments, New Castle, DE, USA). Dynamic measurements were performed from 25 to 600 °C at a heating rate of 10 °C·min^−1^ by using constant nitrogen flow of 60 mL/min to prevent thermal oxidation processes of the sample. The temperatures at 5%, 10%, 25% and 50% of weight loss were calculated. Additionally, isothermal measurements were performed at 150 °C in air to set the mixing time for the preparation of the composites.

#### 2.4.3. Water Absorption and Thickness Swelling

Water absorption and thickness swelling of the biocomposites were determined by immersion of the specimens vertically in distilled water at 25 °C for 24 h (ASTM D570-98) [22]. First, rectangular specimens (24 × 12 × 2.1 mm^3^) were cut from tensile testing fracture specimens and air-dried at 60 °C for 24 h, cooled in a desiccator and weighed (conditioned weight). Then, samples of the biocomposites were soaked in water for 24 h and wiped with paper to remove the excess of water on the surface of the specimens before weighing (wet weight) at fixed time intervals. Three specimens were tested with an analytical balance of 0.1 mg precision and the average and standard deviation were reported. The percentage of water absorption (WA in %) was calculated using Equation (1):

(1) $$\mathrm{WA}\;\left(\%\right)=\frac{\mathrm{wet}\;\mathrm{weight}-\mathrm{conditioned}\;\mathrm{weight}}{\mathrm{conditioned}\;\mathrm{weight}}\times 100$$

And the percentage of thickness swelling was calculated using Equation (2):

(2) $$\mathrm{TS}\;\left(\%\right)=\frac{T_{t}-T_{o}}{T_{0}}\times 100$$

where TS (%) is the percentage of thickness swelling, T_t_ is the thickness at time t, and T_0_ is the initial thickness at t = 0.

#### 2.4.4. Mechanical Properties

The tensile properties of the biocomposites (Young’s modulus, tensile strength, and elongation at break) were evaluated using a tensile test according to the ISO 527 [23] standard with a universal testing machine model 3365 (Instron, Norwood, MA, USA) and controlled by Bluehill Lite software developed by Instron (Norwood, MA, USA). The initial length of the test specimens was 25.4 mm and a cross-head speed of 10 mm/min was used. The number of tested specimens for the mechanical properties was 5 for average calculations.

#### 2.4.5. Field Emission Scanning Electron Microscopy (FESEM)

The polymer/CF interface was analyzed by scanning electron microscopy of the fractured surface of the composites. The microphotographs were taken with a Carl Zeiss Ultra Plus field emission scanning electron microscope (FESEM, Oberkochen, Germany) equipped with an energy dispersive X-ray spectrometer (EDXS). Prior to FESEM analysis, samples were Au-coated.

## 3. Results

### 3.1. Density

**Figure 3:**
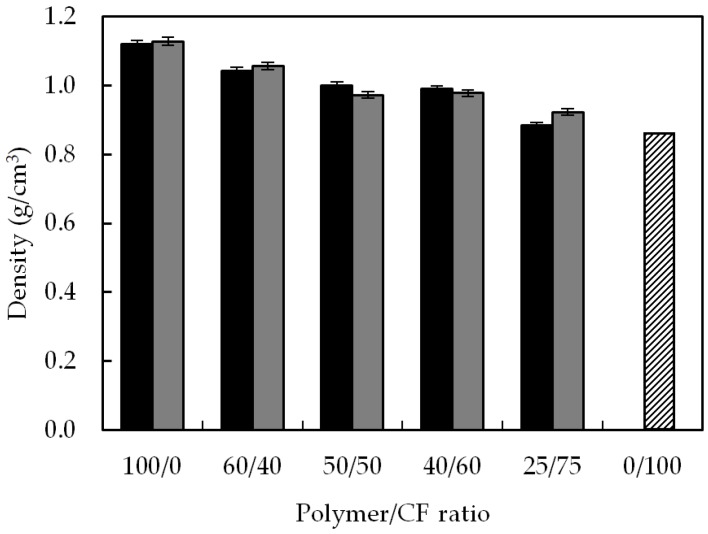
Density of the TPUU-SS/CF (black), TPUU/CF (gray) biocomposites and CF (0/100) as reference.

In Figure 3 the density of neat polymers, CFs and the corresponding biocomposites is shown. The densities of the TPUU-SS and TPUU are 1.120 and 1.128 g/cm^3^, respectively, and the reference density of the CFs was taken as 0.85 g/cm^3^, the average of the reported values [24,25] ~24% less dense than the neat polymers. The density of the biocomposites ranged between the density of the polymers and the CFs and decreased as the CF content was increased. In Table 1, the percentage of the reduction compared to their respective neat polymer is shown, reaching up to 21.4% for TPUU-SS and 18.2% for TPUU in the best case. Both series show identical trend and similar relative weight reductions leading to lightweight biocomposites.

### 3.2. Thermogravimetric Analysis (TGA)

**Figure 4:**
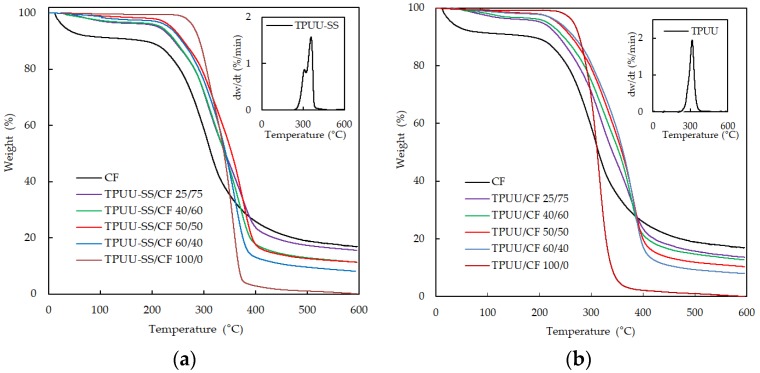
TGA curves of (**a**) TPUU-SS/CF and (**b**) TPUU/CF biocomposites containing different polymer/CF ratio and CF.

The thermogravimetric analysis of the biocomposites is shown in Figure 4. In the case of CFs, which is shown in Figure 4a,b, three weight-loss steps were observed. In the first stage, from 50 °C to 250 °C, the weight loss is due to the evaporation of water molecules absorbed by the hydrophilic groups of the CFs, 40% of total domains, mainly due to the presence of amino acids such as cysteine and serine [26,27]. The second weight loss, between 250 °C and 400 °C, shows a higher rate and is related to the chemical degradation associated with the destruction of disulfide bonds and the elimination of H_2_S originating from amino acid cysteine [28]. The third weight loss occurred from 400 °C onwards and it is associated with the decomposition of the remaining structure. The thermal degradation behavior for the TPUU-SS and TPUU is complex because their structure combines both polyols and diisocyanates whose individual degradation residues may react among them. A minor mass loss of less than 2% due to the water loss from 25 °C to 265 °C for TPUU-SS and from 25 °C to 250 °C for TPUU was registered. The primary mass loss step was much larger and occurred at 375 °C and 360 °C for TPUU-SS and TPUU, respectively, and is related to the decomposition of the soft and hard segments of the polyurethane [29,30,31]. Because of the complex structure of the poly(urea urethane)s, the degradation results in a complicated mixture of products that is often difficult to interpret. The thermal degradation and stability of poly(urea urethane)s depend on the building blocks, synthesis method and reaction conditions [32]. The derivative thermogravimetric curves of TPUU-SS and TPUU are shown in the upper right corner of Figure 4a,b, respectively. In the case of TPUU-SS, the degradation rates of both soft and hard segments are slightly separated. The final mass loss occurred for temperatures above ~400 °C and is related to the total degradation of the polyurethanes [29]. Biocomposites containing different CF loadings show intermediate mass loss between the mass loss of CFs and that of neat polymers, showing inferior thermal stability than feathers between room temperature and ~300 °C. Then, all the mass loss curves of the biocomposites cross the mass loss curves of the polyurethanes between 310 °C–340 °C for TPUU-SS (Figure 4a) and 280 °C–300 °C for TPUU (Figure 4b). At temperatures above ~400 °C, CF showed the highest char level even if it started to degrade at a lower temperature than biocomposites. The residual mass at 600 °C decreased as polymeric matrix content increased with lowest value for pure elastomers (see Table 2). The enhancement of char formation is probably due to a higher heat resistance of the keratinous structure of feathers. For pure CFs, 16.9 wt % of carbonized residue was left, similar to previous reports [33].

In Table 2, the 5%, 25% and 50% weight-loss temperatures of all the samples shown in Figure 4 are detailed.

**Figure 5:**
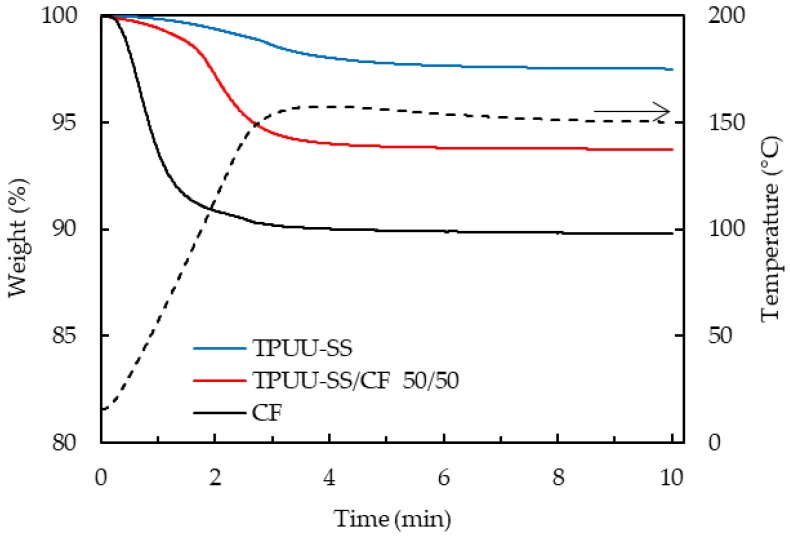
TGA isothermal curves of CF, TPUU-SS/CF (50/50) and TPUU-SS.

In order to verify that biocomposites were thermally stable at 150 °C for 5 min during the consolidation step of their manufacturing process, TGA isothermal curves under oxidative conditions were performed. Initially, a ramp from 25 °C to 150 °C was set, followed by an isotherm during 10 min. In Figure 5 TGA isothermal curves of CF, TPUU-SS and TPUU-SS/CF (50/50) are shown. After the initial weight losses due to moisture evaporation, all materials are thermally stable at 150 °C for at least 7.5 min.

### 3.3. Water Absorption

**Figure 6:**
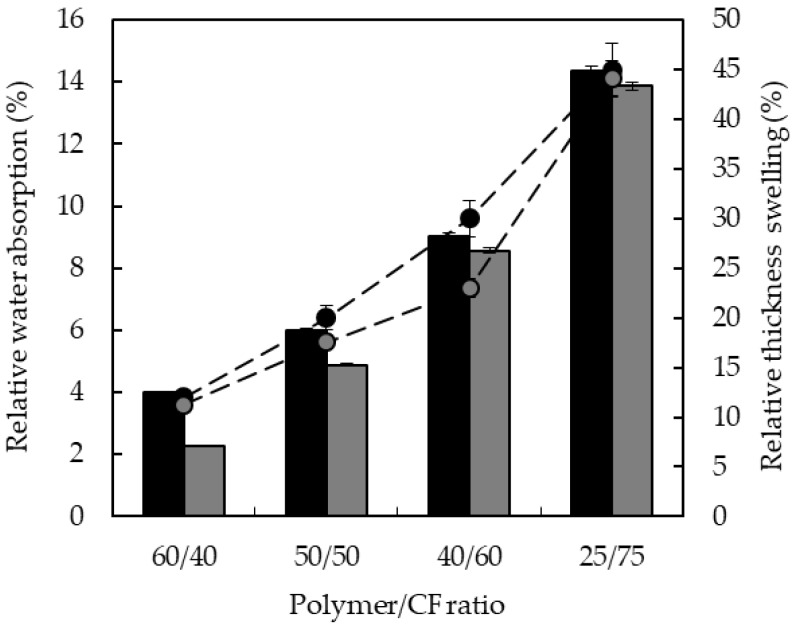
Relative water absorption (vertical bars) and relative thickness swelling (circles) of TPUU-SS (black) and TPUU/CF (gray) biocomposites.

In Figure 6 the relative water absorption and the relative thickness swelling of the biocomposites after 24 h of immersion in water are shown. These values were calculated by dividing the corresponding values with those of the neat elastomers. We observed that, by increasing the content of feathers from 40 to 75 wt %, the relative water absorption increased from 4.00% to 14.3% for TPUU-SS and from 2.2% to 13.8% for TPUU and the relative thickness swelling increased from 11% to 44% for both series of biocomposites. Such increase in the water uptake could be attributed to the high content of hydrophilic amino acids in the keratin structure [34]. Along the whole series, TPUU-SS/CF biocomposites show slightly higher water absorption than TPUU/CF biocomposites, decreasing such difference with the increase of the amount of CFs in the formulation. This observation is likely related to the higher electronegativity of the sulfur compared to carbon and hence has a major affinity to water molecules.

### 3.4. Mechanical Properties of the Biocomposites

**Figure 7:**
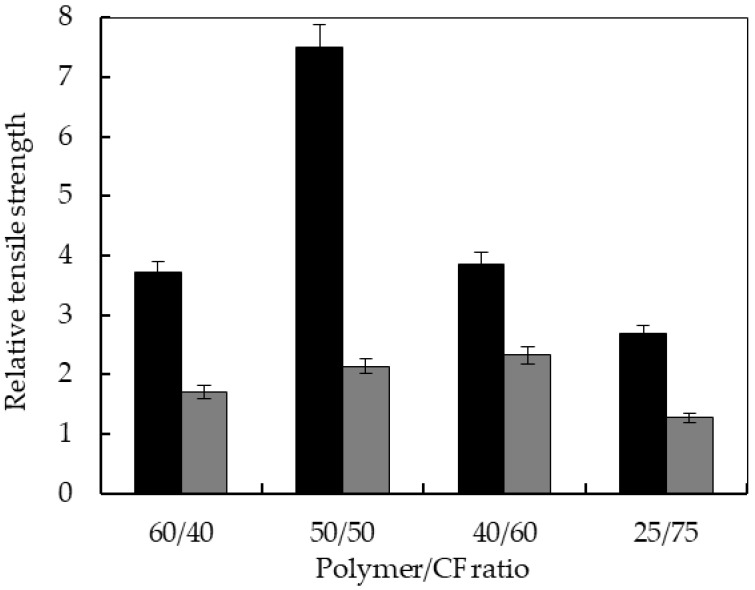
Relative tensile strength of TPUU-SS/CF (black) and TPUU/CF (gray) biocomposites.

**Figure 8:**
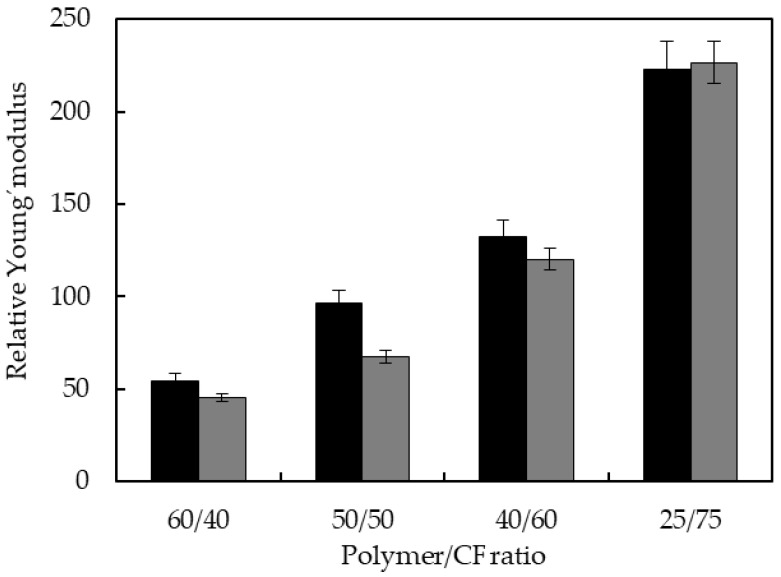
Relative Young’s modulus of TPUU-SS/CF (black) and TPUU/CF (gray) biocomposites.

**Figure 9:**
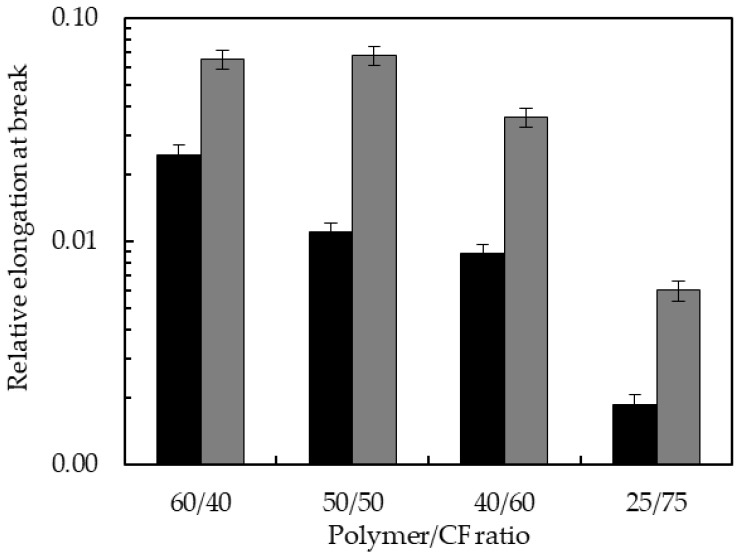
Relative elongation at break of TPUU-SS/CF (black) and TPUU/CF (gray) biocomposites.

The mechanical properties (Young’s modulus, tensile strength and elongation at break) of TPUU-SS/CF and TPUU/CF biocomposites are presented in Figure 7, Figure 8 and Figure 9. Even if both elastomers were prepared from the same polyol precursor, TPUU and TPUU-SS have distinct molecular weight distribution and chemical composition due to the nature of the chain extender used in each case. As a consequence, starting mechanical properties of TPUU and TPUU-SS were significantly different (see Table 3). The absolute values of tensile strength and Young’s modulus of TPUU-SS are lower than TPUU, whereas the elongation at break is two-fold greater. In order to quantify the effect that the presence or not of disulfide moieties in the elastomer might have on the mechanical properties of the biocomposites, relative values were determined. These relative values of the mechanical properties were obtained by the ratio between the properties of the biocomposites and the neat elastomers.

Figure 7 shows the relative tensile strength of TPUU-SS/CF and TPUU/CF biocomposites. All biocomposites showed a higher relative tensile strength than the corresponding pristine elastomer. The maximum relative tensile strength was observed for TPUU-SS/CF (50/50) with a value of 7.5, while for TPUU/CF biocomposites the maximum relative tensile strength corresponded to (40/60) composition with a value of only 2.3. These results illustrate a stronger bonding between the CFs and the TPUU-SS matrix. For all the ratios, improvement in the relative tensile strength is higher when the polyurethane contains disulfide linkages, reinforcing the idea that the TPUU-SS presents a better adhesion towards the structure of the keratin. The lowest relative tensile strength was found when the CF content was 75 wt %, likely due to the low polymer content and hence low adhesion between the matrix and the fibers.

In Figure 8 the relative Young’s moduli of the biocomposites are presented. For both series of biocomposites, the Young’s modulus increased progressively as CF content increased, obtaining biocomposites with high stiffness and relative Young’s moduli 225 fold larger.

Compared to biocomposites based on TPUU matrices, most of the biocomposites (60/40, 50/50, 40/60) containing S–S linkages have larger relative Young’s modulus. For both polyurethane/CF (25/75) biocomposites, the values of Young’s modulus were similar in both series, i.e., the stiffness was given largely by the high CF content.

Figure 9 shows in logarithm scale the relative elongation at break of the different biocomposites. As it is common for fiber-reinforced composites, the elongation at break decreased as fillers were added to the neat polyurethane matrices. The clearest case is the TPUU-SS/CF series, diminishing the elongation at break from 0.025 to 1.8 × 10^−3^ times the elongation at break of neat TPUU-SS. In the TPUU/CF series elongation at break was also drastically reduced with relative elongation at break in the range of 6 × 10^−2^–6 × 10^−3^ of the initial value of neat TPUU. These results are in agreement with results observed in several CFs reinforced biocomposites such as thermoplastic PU composites [35] and polybutylene adipate terephthalate, a thermoplastic matrix with elongation at break close to 550% [4]. Definitely, CFs stiffened the biocomposites and reduced the initial high ductility of the neat TPUUs. A lower ductility of the TPUU-SS/CF biocomposites is expected since a larger entanglement at molecular level between CFs and the TPUU-SS is likely to occur.

In short, Young’s moduli and tensile strength of biocomposites reinforced with chicken feathers increased significantly compared to neat elastomers obtaining biocomposites with much higher strength and stiffer. In addition, biocomposites based on elastomers containing disulfide bonds showed enhanced relative mechanical properties, attributable to a higher adhesion between the feathers and disulfide bonds containing polymer matrix. The maximum and minimum relative tensile strength of TPUU-SS/CF were at 50 and 75 wt %, respectively, leading to the conclusion that there was not enough polymer material at the higher level of feathers to provide good bonding between the feathers and the matrix.

**Figure 10:**
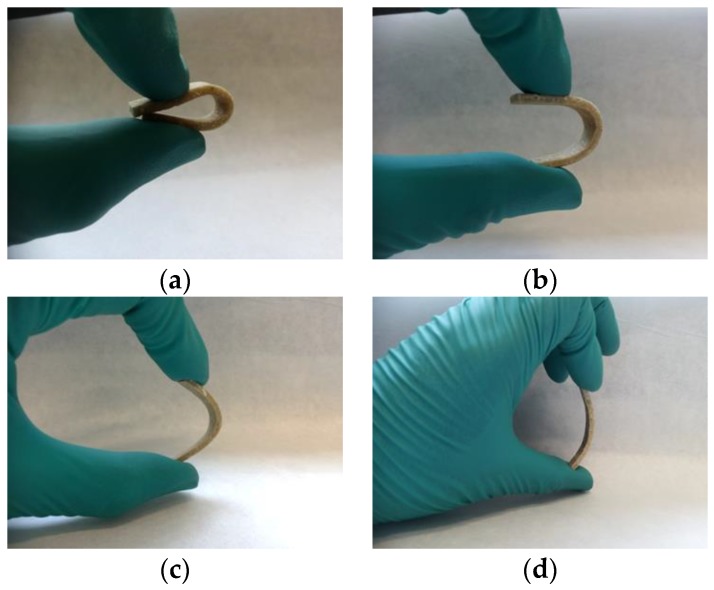
Maximum bending of keratin-based TPUU-SS/CFs biocomposites containing different loadings of CF: (**a**) 40 wt %, (**b**) 50 wt %, (**c**) 60 wt % and (**d**) 75 wt %.

In order to visualize the effect of the CFs on the flexibility of TPUU-SS/CF biocomposites, rectangular samples were manually bended at the maximum extend before breaking. In Figure 10, the approximately maximum bending of biocomposites containing 40, 50, 60 and 75 wt % of CFs can be observed. Undoubtedly, all biocomposites showed certain bending degree which decreased gradually as CFs loading increased.

### 3.5. Morphology of the TPUU/CF and TPUU-SS/CF Biocomposites

**Figure 11:**
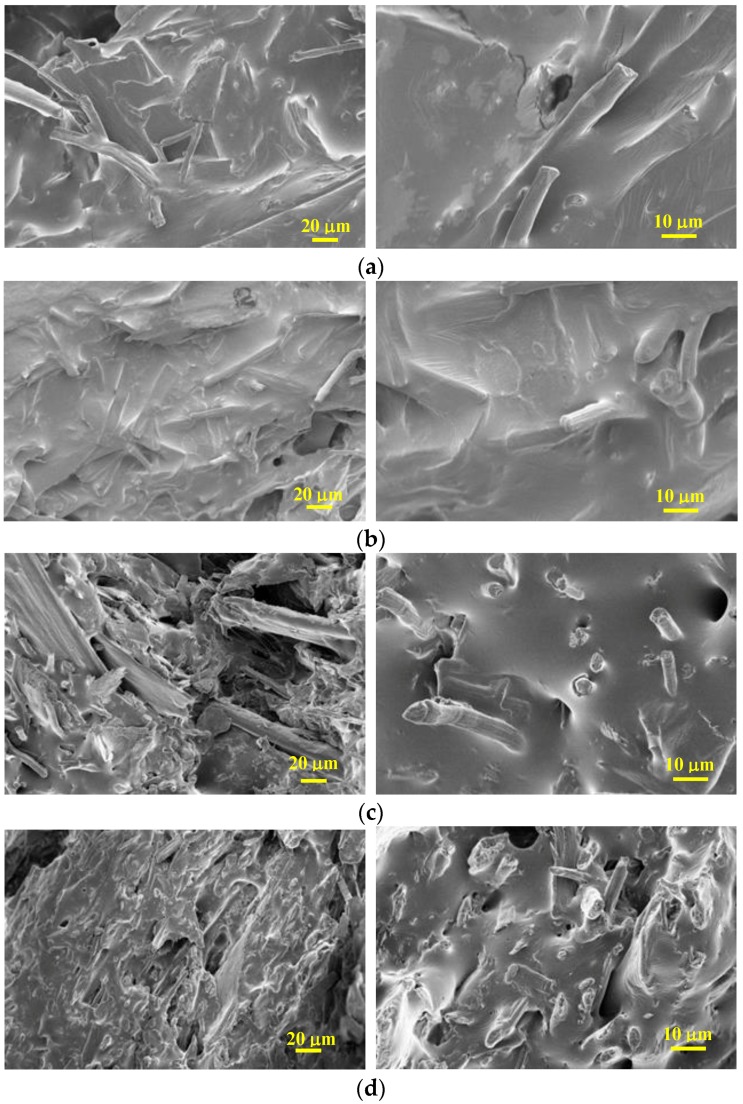
Field Emission Scanning Electron Microscope (FESEM) images of (**a**) TPUU-SS + 40 wt % CF; (**b**) TPUU-SS + 60 wt % CF; (**c**) TPUU + 40 wt % CF and (**d**) TPUU + 60 wt % CF.

The morphology of fractured surfaces of TPUU-SS/CF and TPUU/CF biocomposites with polymer/CF ratios of 60/40 and 40/60 was recorded using FESEM (Figure 11). Each sample was observed with two different magnifications, 1.000× and 3.000×. In Figure 11a,b the biocomposites based on TPUU-SS containing 40 wt % and 60 wt % of CF are shown. The ground CF fibers were well dispersed and completely covered by the polymer matrix, which consequently imparts better fiber–matrix adhesion as it was observed in tensile properties. In Figure 11c,d the FESEM micrographs show the fractured surface of TPUU/CF (60/40) and (40/60), respectively, and two opposite morphological situations can be described: (a) domains with individual fibers finely dispersed and covered with a thin film of TPUU polymer with a significant interaction between the fiber and the matrix, and (b) domains with poor interaction between the TPUU and the CF, showing uncovered fibers and some holes from fiber pull-outs. The adhesion between the CFs and the matrix is lower in polyurethanes lacking disulfide bonds which is in agreement with the results observed after the mechanical characterization of the biocomposites. Clearly, the matrix containing disulfide linkages interact better with chicken feathers.

## 4. Conclusions

In this work, we comparatively characterized biocomposites made of CFs and two types of poly(urea-urethane) elastomers, TPUU-SS and TPUU. These elastomers were synthesized from the same polyol but differ in the type of chain extender used: bis(4-aminophenyl) disulfide and the sulfur-free counterpart bis(4-aminophenyl) methane, respectively. The selection of disulfide containing polymer was motivated by the aim of increasing the compatibility of classical poly(urea-urethanes) towards CFs. This idea was based, on the one hand, on the incorporation in the elastomer structure of the same chemical moieties which are present in the keratin. On the other hand, aromatic disulfide linkages provide a new dynamic mechanism to the polymer system that might assist in the cohesion of the composite in combination with hydrogen-bonds.

A set of characterizations of the biocomposites, such as density, relative water absorption and relative thickness swelling showed similar trends in both series of biocomposites. However, mechanical characterization of the materials pointed out distinct effect. Relative mechanical properties of both formulations were compared under the same testing conditions and fiber content (40%, 50%, 60% and 75%). The results demonstrated that the addition of the CFs fibers had indeed a greater positive effect on the mechanical properties of the biocomposites made of TPUU-SS. The case of biocomposites with 50% CFs loading is especially remarkable: 7.5-fold versus 2.3-fold improvement on relative tensile strength were recorded for the disulfide-containing and sulfur-free counterpart, respectively. This observation leads us to the conclusion that the TPUU-SS presents better interfacial compatibility towards CFs, presumably due to the proposed hypothesis of better cohesion of the polymer blends. This conclusion is also supported by morphological analyses of the fractured surfaces by FESEM. Only in the case of disulfide-containing formulations CF fibers were fully impregnated, with the absence of voids.

Chicken feathers are produced in large quantities as a by-product at poultry processing plants. They are cheap, low density, abundantly available and renewable, delivering strong and stiff fibers, intrinsic characteristics of vital importance for the valorization of this waste as reinforcing material in composite materials. Here, we demonstrate that flexible poly(urea-urethane) composites with high CFs loading can be prepared by conventional polymer processing techniques and that interfacial adhesion can be optimized by incorporating matrices with aromatic-disulfide linkages.

## Figures and Tables

**Table 1:** Percentage of density reduction of the biocomposites.

Matrix	Matrix/CF Ratio	% of Density Reduction Compared to Neat Polymer Matrix
	100/0	-
	60/40	7.1
TPUU-SS/CF	50/50	10.7
	40/60	11.6
	25/75	21.4
	100/0	-
	60/40	6.5
TPUU/CF	50/50	13.8
	40/60	13.3
	25/75	18.2

**Table 2:** Thermal characterization of TPUU-SS/CF, TPUU/CF and CF samples.

Sample	Polymer/CF Ratio	T (5%) (°C)	T (25%) (°C)	T (50%) (°C)	% Residual Mass after 600 °C
TPUU-SS/CF	100/0	280.31	310.98	339.04	0.13
	60/40	232.36	301.98	342.29	8.14
	50/50	237.38	306.93	354.02	11.02
	40/60	217.24	293.96	340.27	11.36
	25/75	211.79	293.90	351.38	15.59
TPUU/CF	100/0	265.78	294.85	311.36	0.01
	60/40	237.98	314.12	361.63	8.03
	50/50	234.87	310.09	359.27	10.12
	40/60	215.29	299.57	353.56	12.72
	25/75	204.08	289.78	341.17	13.62
CF	0/100	30.76	268.13	313.94	16.92

**Table 3:** Tensile properties of neat polymers and CFs reinforced biocomposites.

Type of Matrix	Polymer/CF	Young’s Modulus (MPa)	Tensile Strength (MPa)	Elongation at Break (%)
TPUU-SS	100/0	1.24	1.03	1030.25
	60/40	67.45	3.82	25.25
	50/50	120.12	7.73	11.37
	40/60	164.05	3.98	9.11
	25/75	276.06	2.78	1.92
TPUU	100/0	1.81	4.80	450.10
	60/40	81.61	8.18	29.48
	50/50	122.04	10.28	30.73
	40/60	216.57	11.16	16.21
	25/75	407.83	6.09	2.71
